# What is the Optimum Pattern of Pressurisation to Gain Maximum Penetration of Methylmethacrylate Cement into the Reamed Acetabulum?

**DOI:** 10.7759/cureus.6654

**Published:** 2020-01-14

**Authors:** Brett Rocos, Michael R Whitehouse

**Affiliations:** 1 Orthopaedics, North Bristol National Health Service Trust, Bristol, GBR; 2 Musculoskeletal Research Unit, University of Bristol, Bristol, GBR

**Keywords:** hips, cement, arthroplasty

## Abstract

Introduction

The cemented polyethylene cup has remained the standard acetabular implant for 50 years although there has been little research into cementing techniques. In the past, cement was previously inserted by sequential manual pressurisation (thumbing) but this technique was prone to contamination of the cement leading to weakening of fixation. In recent times, third-generation techniques using sealed pressurisation with rim preparation have been espoused with similar results. We were interested in establishing whether repeated cycles of compression of cement allowing adequate time for relaxation increases its depth of penetration, and the optimum period of relaxation required to achieve this goal.

Method

A single mix of polymethylmethacrylate cement at dough stage was inserted into a model of the reamed acetabulum. Cyclical pressurisation of the cement with 50 N followed nine different patterns to simulate thumbing, constant pressure, and the application of a sealed and unsealed acetabular cup implant.

Results

A constant load was as effective as all variations of repeated cycles of load and relaxation except for 50 N pressure applied for four seconds with four second intervals. A four second interval of relaxation achieved significantly more penetration than five or three seconds.

Following two minutes of constant pressure, the application of a sealed or unsealed thrust of the plunger had no effect on cement penetration.

Conclusion

This study suggests that optimal polymethylmethacrylate cement penetration into the acetabulum occurs with cycled application of load for four seconds followed by four seconds of relaxation. The subsequent pressurisation with either a flanged or unflanged acetabular implant does not appear to improve cement penetration.

## Introduction

The cemented polyethylene cup was first implanted in 1962, and since then has remained a popular option for acetabular reconstruction [1-5]. The cemented cup tends to demarcate radiologically at the cement-bone interface and, whilst this is usually not progressive, uncemented devices are now more widely used despite their higher rates of revision [1,5]. Although fixation of the femoral component has been improved by compression of cement at low viscosity into restricted clean dry bone, there has been disproportionately little research on acetabular cementing technique. Keyholes were adopted to promote interference between cement and bone, and cement penetration into these enhanced by gunning in low viscosity cement at high pressure, and negative pressure from iliac suction and positive pressure from flanged cups have been introduced; however, clinical results have not significantly improved [1,6-11].

The cementing technique aims to increase the penetration of cement in order to enhance the shear strength of the cement-bone interface, and it has been well established that cement penetration is directly related to pressure and inversely to viscosity [7,12,13]. In the femur, cement of low viscosity can be used because the cavity is first conical then tubular, whereas the acetabulum is hemispherical. In contrast, the acetabular hemisphere cannot be restricted and so cement must be inserted as dough to prevent its escape when pressure is applied. When cemented acetabular implants were initially introduced, cement was inserted by repetitive manual pressure (also known as thumbing); however, it was later found that this caused laminations of blood which has the effect of weakening the structural integrity of the interface [14]. This occurs because cement is a viscoelastic material, and as a result its properties change with the properties of the pressure applied. In the clinical setting, Abdulghani showed that this property means that bone cement fails to transmit pressure for more than 30 seconds following the initiation of pressure, and Markolf demonstrated that 89% of cement penetration inserted four minutes after mixing occurs within the first four seconds of a single cycle of pressure [15,16]. In the modern era, maximising cement penetration in order to achieve interlock and reduce the risk of subsequent revision is a central goal. Knowing the above, we were interested in establishing whether repeated cycles of compression of cement in the acetabulum whilst allowing time for relaxation could increase its depth of penetration and the optimum period of relaxation required to achieve this goal by duplicating the ‘thumbing’ technique and extending the period of compression to four seconds (corresponding to the findings of Markolf) whilst varying the periods of relaxation allowed [16]. These patterns of compression were compared with controls of constant pressure both with and without the subsequent compression of a simulated acetabular cup insertion.

We hypothesised that intermittent pressurisation for four seconds with relaxation of two seconds would give rise to optimal penetration when compared to differing relaxation times or constant pressure because of the excellent results historically achieved with the thumbing technique.

## Materials and methods

A single mix of methylmethacrylate cement (Palacos R 40, Hereus Medical, Wehrheim, Germany) was prepared in a fume cabinet in a cement gun (Palamix vacuum mixing system, Heraeus Medical, Wehrheim, Germany) following the manufacturer’s instructions. A vacuum system was not used. The cement was allowed to reach the dough stage which took 120 seconds from commencement of mixing at a temperature of 21.5 degrees Celsius.

The cement was injected into the centre of an BS ISO 5833: 2002 (E) compliant polytetrafluoroethylene model using the cement gun. This model is used by manufacturers to standardise the dough time and measure the viscosity of methylmethacrylate cement during curing [17].

**Figure 1:**
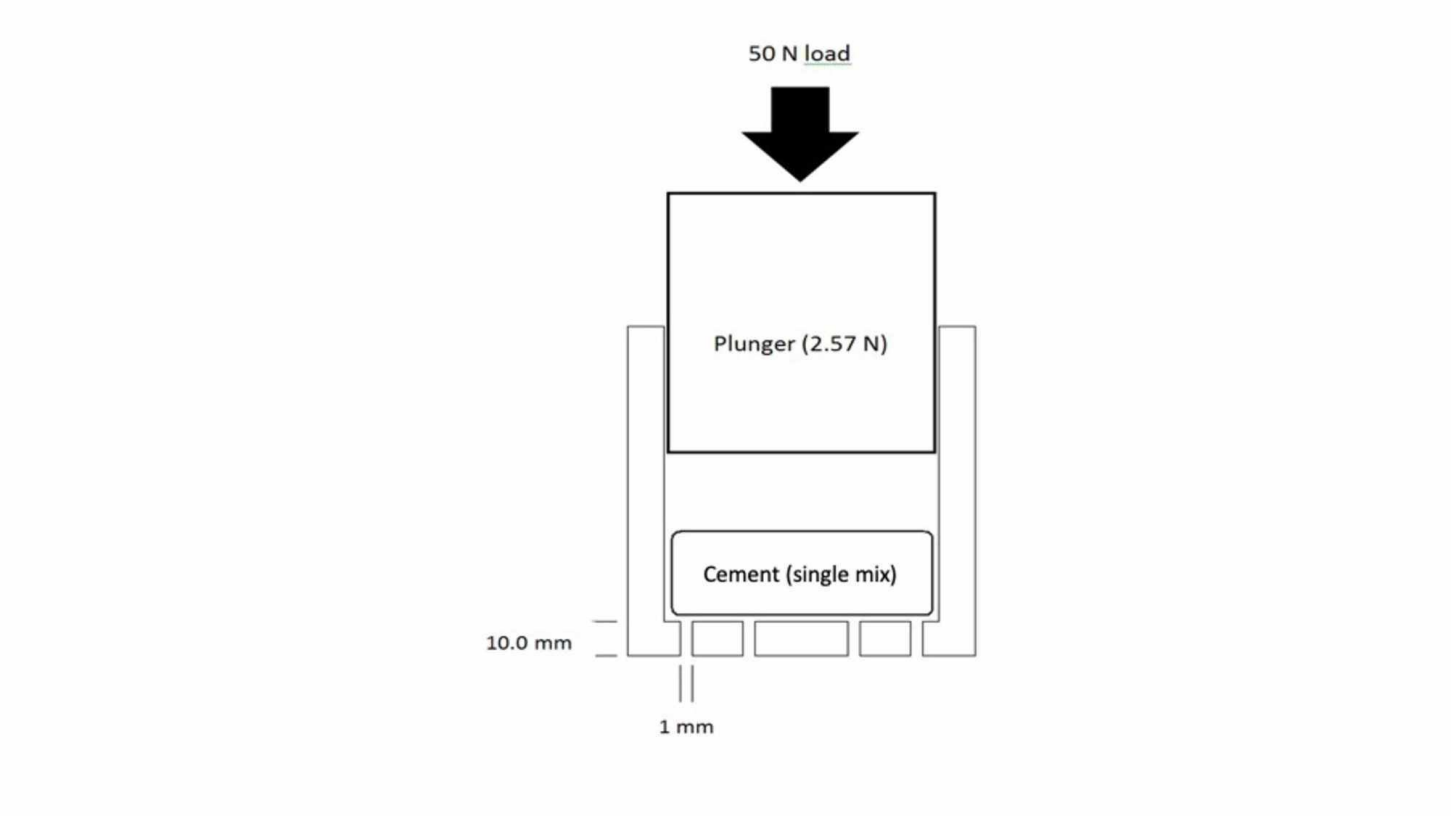
The BS ISO 5833: 2002 (E) used in the experimental model

The cement was not redistributed around the base of the mould. The mould plunger was then inserted and a load of 50 N was applied (Figure 1) following five different patterns:

1. Pressure applied for four seconds at intervals of one, two, three, four or five seconds for two minutes in total to simulate the ‘thumbing’ technique. The relaxation intervals between pressure application were selected based upon the senior author’s experience of using the technique in vivo.

2. Pressure applied for 30 seconds at four second intervals for two minutes.

3. Constant pressure for two minutes.

4. Constant pressure for two minutes, 150 seconds of curing then a 10 second manually applied sealed plunger thrust to simulate flanged cup insertion.

5. Constant pressure for two minutes, 150 seconds of curing then a 10 second unsealed plunger thrust to simulate unflanged cup insertion.

Immediately after the pressurisation sequence was complete, the depth of cement penetration into the pores was measured. Each pattern of pressurisation yielded four results. Each test was repeated at least four times, yielding a minimum of 16 results for each pressure pattern. Data were analysed by a one-way ANOVA test using SPSS v. 20 (IBM SPSS Statistics for Windows, Version 20.0. IBM Corp. Armonk, NY).

## Results

**Figure 2:**
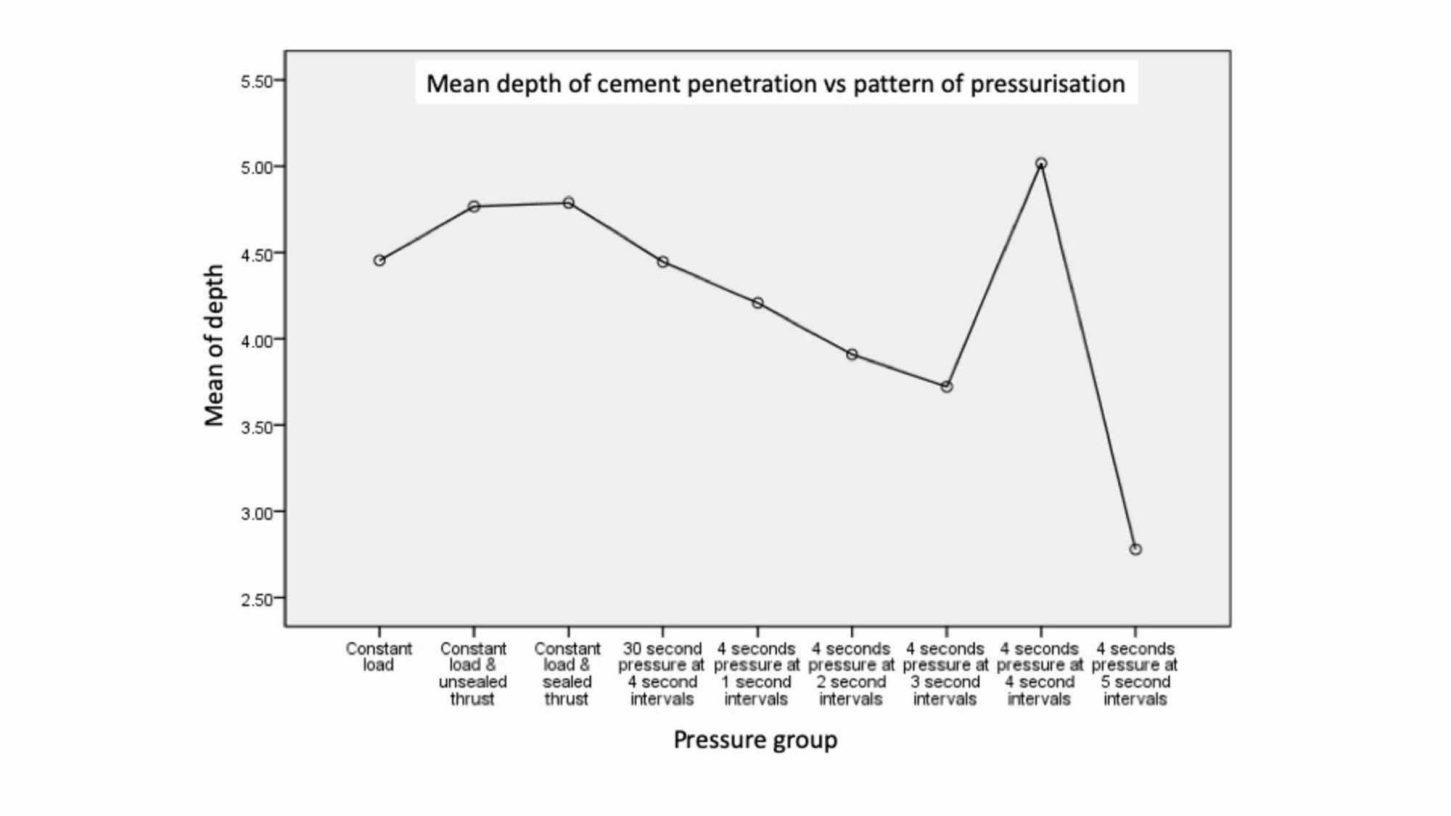
The depth of cement penetration into the model for each pattern of pressurisation

Constant load was as effective as all variations of repeated cycles of load and relaxation except for 50 N pressure applied for four seconds with four second intervals. A four second interval of relaxation achieved significantly more penetration than five or three seconds (p<0.005) (Figure 2).

Following two minutes of constant pressure, the application of a sealed or unsealed thrust of the plunger (representing cup insertion) after 150 seconds of cement polymerisation had no effect on cement penetration.

## Discussion

These results show that intermittent pressurisation of the cement with four seconds of force at four second intervals for two minutes provides as much cement penetration as constant pressure for two minutes followed by a thrust with a sealed or unsealed cup.

The data from groups 4 and 5 show that additional pressurisation after two minutes following dough time makes no significant difference to the penetration of cement into the acetabular cancellous pores. These results go on to suggest that pressurisation with a cup following adequate initial cement pressure does not enhance penetration. Therefore, it is the initial pressurisation strategy that is responsible for most, if not all, of the cement penetration into the reamed acetabulum. This is consistent with the observations of Abdulghani et al. who showed that 98% penetration occurred during initial pressurisation, and of Markolf and Amstutz who concluded that 89% of cement penetration occurred within the first four seconds of pressurisation [15,16].

If we take these results in light of the work performed by Ørskov, who showed that a flanged cup does not improve cementation compared with unflanged cup, and Battacharya who showed that the pressure generated within the cement mantle with flanged or unflanged cups (i.e., unsealed or sealed systems) is the same, then it could be postulated that increasing pressure late in the cementation process does not enhance penetration of cement in dough form [10,11].

We are therefore able to suggest that flanges on cups or attempts to create a sealed system during cup pressurisation do not increase penetration into the reamed acetabulum, and that it is the initial pressurisation technique that is one of the principal factors influencing the quality of fixation.

Future work would need to explore the viscoelastic properties of alternative bone cements, and the influence of differing bone quality on the depth of penetration. Preparing cement with the benefit of a vacuum system may also play a role in the mechanical properties of the cement, and subsequent studies would ideally take this into account. Another aspect that subsequent work would need to address would be the impact of blood laminations in the final cement mantle, which would likely influence the in vivo integrity of the fixation, some of which have been tackled with the third-generation techniques [18]. Furthermore, with the developments in implant technology, it may be that some of the findings of studies which underpin this work need revisiting in order to update their findings.

## Conclusions

It seems likely, based on these results, that the ‘thumbing’ technique delivers comparable cement penetration to more contemporary techniques, which would account for the consistent results using the thumbing technique over the last five decades.

Despite the advances seen in third-generation cementing techniques which seek to improve the consistency of cement and pressurisation, our results suggest that the future of cementing in a reamed acetabulum may not lie solely in systems that increase pressurisation but in improving the bone into which the cement is introduced. Intraoperatively, this means that grafting the fovea and gaining access to cancellous bone around the periphery using burrs or drill holes is likely to improve the longevity and quality of fixation.
